# Anti-Factor H Antibody Reactivity in Young Adults Vaccinated with a Meningococcal Serogroup B Vaccine Containing Factor H Binding Protein

**DOI:** 10.1128/mSphere.00393-19

**Published:** 2019-07-03

**Authors:** Kelsey Sharkey, Peter T. Beernink, Joanne M. Langley, Soren Gantt, Caroline Quach, Christina Dold, Qin Liu, Manuel Galvan, Dan M. Granoff

**Affiliations:** a Center for Immunobiology and Vaccine Development, University of California San Francisco Benioff Children’s Hospital Oakland, Oakland, California, USA; b IWK Health Centre and the Nova Scotia Health Authority, Canadian Center for Vaccinology at Dalhousie University, Halifax, Nova Scotia, Canada; c BC Children’s Hospital Research Institute, Vancouver, British Columbia, Canada; d McGill University Health Centre Research Institute and CHU Sainte Justine, Montreal, Quebec, Canada; e University of Oxford, Oxford, United Kingdom; f Wistar Institute, Philadelphia, Pennsylvania, USA; g National Jewish Health Complement Laboratory, Denver, Colorado, USA; National Institute of Allergy and Infectious Diseases

**Keywords:** 4C-MenB, Bexsero, FHbp, factor H, MenB-4C, *Neisseria* meningitidis, vaccines

## Abstract

Meningococcal serogroup B (MenB) vaccines contain recombinant factor H binding protein (FHbp), which can complex with complement factor H (CFH) and thereby risk eliciting anti-FH autoantibodies. While anti-FH antibodies can be present in sera of healthy persons, the antibodies are implicated in autoimmune atypical hemolytic uremic syndrome and C3 glomerulopathies. We immunized 120 students with a MenB vaccine (Bexsero). By enzyme-linked immunosorbent assay (ELISA), there were small increases in serum anti-FH levels at 3 weeks postvaccination (geometric mean optical density at 405 nm [OD_405_], 0.54 versus 0.51 preimmunization, *P ≤ *0.003 for each schedule tested). There was a similar small increase in anti-FH antibody levels in a second historical MenB study of 20 adults with stored paired preimmunization and postimmunization sera (*P = *0.007) but not in three other studies of 57 adults immunized with other meningococcal vaccines that did not contain recombinant FHbp (*P = *0.17, 0.84, and 0.60, respectively). Thus, humans vaccinated with MenB-4C develop small increases in serum anti-FH antibody reactivity. Although not likely to be clinically important, the data indicate a host response to FH. In the prospective MenB study, three subjects (2.5%) developed higher anti-FH titers postimmunization. The elevated titers returned to baseline within 3 to 4 months, and none of the subjects reported adverse events during the follow-up. Although anti-FH antibodies can decrease FH function, the postimmunization sera with high anti-FH antibody levels did not impair serum FH function as measured using a hemolytic assay. Thus, while additional studies are warranted, there is no evidence that the anti-FH antibodies elicited by MenB-4C are likely to cause anti-FH-mediated autoimmune disorders. (This study has been registered at ClinicalTrials.gov under registration no. NCT02583412.)

**IMPORTANCE** Meningococci are bacteria that cause sepsis and meningitis. Meningococcal species are subdivided into serogroups on the basis of different sugar capsules. Vaccines that target serogroup A, C, Y, and W capsules are safe and highly effective. New serogroup B (MenB) vaccines target a bacterial protein that can bind to a blood protein called complement factor H (FH). While serogroup B vaccines appear to be safe and effective, there is a theoretical risk that immunization with a bacterial protein that binds host FH might elicit anti-FH autoantibodies. Autoantibodies to FH have been detected in healthy persons but in rare cases can cause certain autoimmune diseases. We found small and/or transient increases in serum antibody to FH after MenB immunization. While no serious adverse events were reported in the subjects with elevated anti-FH titers, since onset of autoimmune disease is a rare event and may occur months or years after vaccination, additional, larger studies are warranted.

## Abstract (precis)

Meningococci are bacteria that cause sepsis and meningitis. Meningococcal species are subdivided into serogroups on the basis of different sugar capsules. Vaccines that target serogroup A, C, Y, and W capsules are safe and highly effective. New serogroup B (MenB) vaccines target a bacterial protein that can bind to a blood protein called complement factor H (FH). While serogroup B vaccines appear to be safe and effective, there is a theoretical risk that immunization with a bacterial protein that binds host FH might elicit anti-FH autoantibodies. Autoantibodies to FH have been detected in healthy persons but in rare cases can cause certain autoimmune diseases. We found small and/or transient increases in serum antibody to FH after MenB immunization. While no serious adverse events were reported in the subjects with elevated anti-FH titers, since onset of autoimmune disease is a rare event and may occur months or years after vaccination, additional, larger studies are warranted.

## INTRODUCTION

Serogroup B meningococci have a capsular polysaccharide that is antigenically similar to sialic acid polysaccharides expressed by human cells (1). Because of limited immunogenicity of this bacterial polysaccharide “self-antigen” and the risk of eliciting cross-reacting autoantibodies, vaccine developers looked beyond the capsule for alternative vaccine antigens to promote protective immunity (reviewed in reference 2). Among the various protein antigens investigated, factor H (FH) binding protein (FHbp) elicited broad serum bactericidal activity and ultimately became an important antigen in two recently licensed meningococcal vaccines for prevention of serogroup B disease. These vaccines are referred to as MenB-4C (Bexsero; GlaxoSmithKline) (so named because the vaccine contains four protective component antigens) and MenB-FHbp (Trumenba; Pfizer), which contains only FHbp.

FH is a complement-downregulating protein present in high concentrations in serum (3). FH binds to host cells to inhibit amplification of complement activity and thus helps to prevent damage to host tissues when the complement cascade is activated. Meningococci have evolved mechanisms to recruit human FH to the bacterial surface that enable the organism to downregulate complement activation and evade complement-mediated bacteriolysis (4, 5). Among the several meningococcal FH ligands (4), FHbp is the most important and is highly specific for human FH (6) and for some nonhuman primate FH (7).

The FHbp antigen in meningococcal serogroup B vaccines can form a complex with FH that affects recognition of the bacterial antigen by the host. For example, studies in human FH transgenic mice and rhesus macaques indicated that binding of FH to the FHbp vaccine antigen decreases anti-FHbp serum bactericidal antibody responses (8–10). In theory, the interaction between host FH and the FHbp vaccine antigen also could elicit serum anti-FH autoantibodies. Experimental support for this hypothesis comes from a MenB-4C immunogenicity study in human FH transgenic mice in which a few mice developed serum IgM anti-FH antibodies (9). Also, in infant rhesus macaques whose macaque FH binds to FHbp similarly to human FH, two MenB-4C-vaccinated animals in each of two studies developed transient serum IgG anti-FH antibodies (11, 52).

Autoantibodies to FH have been causally implicated in rare diseases of complement dysregulation such as autoimmune atypical hemolytic uremic syndrome (aHUS) (12, 13) and C3 glomerulopathies (C3G) (14). There is no evidence to date that humans immunized with serogroup B vaccines are at higher risk of developing these diseases. However, given the plausibility of FHbp elicitation of anti-FH antibodies, we undertook the first study in humans to evaluate anti-FH autoreactivity after vaccination with a meningococcal serogroup B vaccine.

## RESULTS

### Small increases in serum anti-FH reactivity 3 weeks after MenB-4C vaccination.

**FIG 1:**
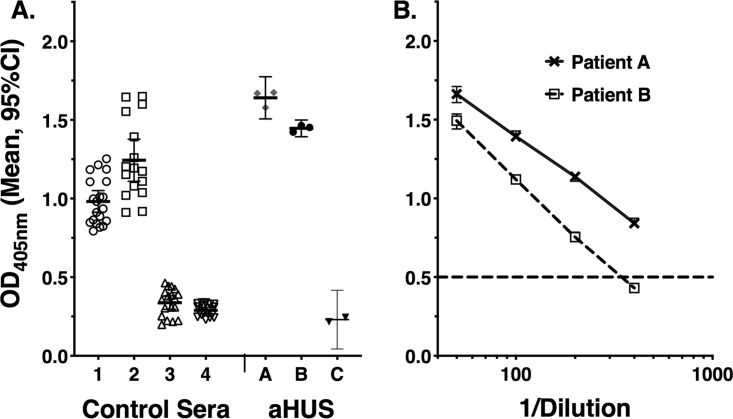
Anti-FH reactivity of control sera assayed on multiple occasions. Assays of the study participants included four control serum samples with high or low anti-FH reactivity. All sera were tested at a 1:50 dilution. (A) OD_405_ values of control sera 1 and 2 with high anti-FH reactivity and control sera 3 and 4 with low reactivity. As a reference, data are shown from assays of sera from three patients with aHUS (patients A and B, with autoimmune aHUS, and patient C, negative for anti-FH antibody and with abnormal FH function, presumably on a genetic basis). (B) Serum anti-FH titers of patients A and B with autoimmune aHUS. The Ig antibodies of patient A and patient B sera were reported to be reactive with the C-terminal and N-terminal portions of the FH molecule, respectively (15). The serum titers were >1:500 (patient A) and 1:345 (patient B) based on the serum dilution calculated to give an OD_405_ intercept of 0.5.

Total (IgG, IgM, and IgA) serum anti-FH reactivity was measured by enzyme-linked immunosorbent assay (ELISA) as described in Materials and Methods. The assays included, as controls, two serum samples from unvaccinated adults with low anti-FH reactivity and two serum samples from adults with high anti-FH reactivity (one from a vaccinated adult and the other from an unvaccinated adult). All sera were assayed at a low (1:50) dilution. In replicate assays performed over 6 months, the mean optical density at 405 nm (OD_405_) values ± standard deviations (SD) determined for the two control sera with low reactivity were 0.34 ± 0.09 and 0.29 ± 0.04, and the values determined for the two control sera with high reactivity were 1.24 ± 0.25 and 0.98 ± 0.15 (Fig. 1A). As a reference, sera from three patients with aHUS were tested. Patients A and B had autoimmune aHUS with mean anti-FH OD_405_ values of 1.64 ± 0.05 and 1.45 ± 0.02. In a previous study, the antibodies in these sera were reported to be reactive with the C-terminal and N-terminal portions of the FH molecule, respectively (15). In repeat assays of these sera performed in our laboratory using serial dilutions, the anti-FH titers were 1:345 and >1:500, respectively (based on the serum dilution with an OD_405_ intercept of 0.5; Fig. 1B). Patient C was negative for anti-FH antibody and had abnormal FH functional activity that was presumed to be genetic in origin (see Fig. 3B).

In the prospective multicenter MenB-4C immunogenicity study, both groups of subjects assigned to either the accelerated or standard vaccination schedule showed small but statistically significant increases in geometric mean anti-FH reactivity 3 weeks after vaccination. The geometric mean OD_405_ for subjects in the accelerated schedule was 0.51 before vaccination compared with 0.54 after vaccination (*P < *0.0001). Similarly, for the standard vaccination schedule, the respective geometric means were 0.51 and 0.54 (*P = *0.003). As described below, 3 of the 120 subjects showed elevated anti-FHbp titers in the postimmunization sera. However, even omitting the data from these three subjects, the increases in geometric mean OD_405_ values after vaccination of the remaining subjects were still statistically significant (*P = *0.0001 and *P = *0.005 for the two vaccine schedules). Thus, while the absolute increases in antibody binding OD_405_ values after immunization were small, as described below, the null hypothesis of no change could be rejected with high probability. Therefore, we can infer a weak immune response to FH in the subjects vaccinated with MenB-4C.

**FIG 2:**
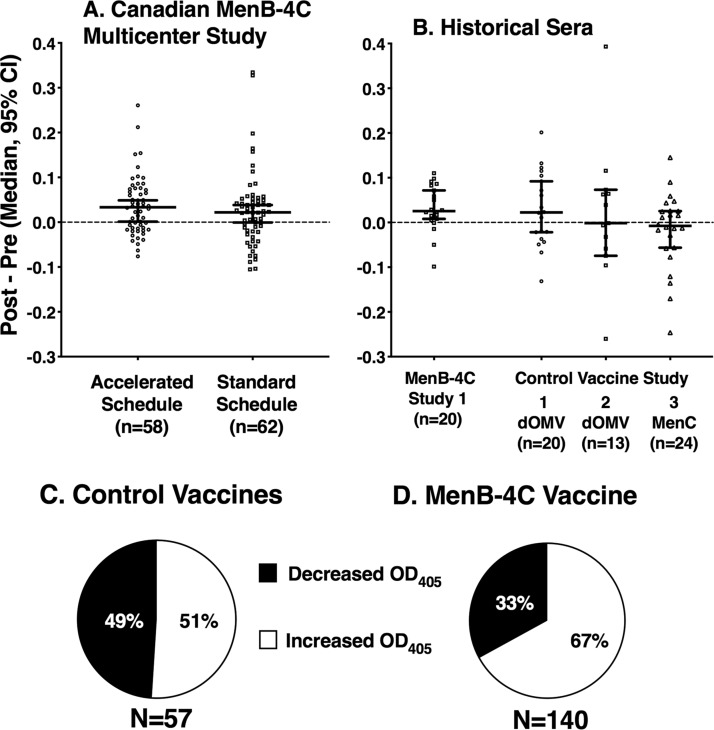
Changes in serum anti-FH reactivity after MenB-4C vaccination. (A and B) Each data point represents the difference between the post-OD_405_ and pre-OD_405_ values determined for a vaccinated subject. Error bars represent the median values for each group and 95% confidence intervals. (A) Prospective Canadian study. Subjects assigned to either MenB-4C vaccination schedule showed small but statistically significant increases in anti-FH reactivity 3 weeks after vaccination (the lower limits of the 95% confidence interval [95% CI] are not below 0; *P* = 0.0001 and 0.028, in the accelerated and standard vaccination schedules, respectively, by one-sample *t* test). (B) Changes in anti-FH reactivity in historical paired pre- and postimmunization sera. There was an increase in serum anti-FH binding activity after MenB-4C vaccination, comparing the pre- and postimmunization sera (*P* = 0.007 by one-sample *t* test), but not after vaccination with other meningococcal vaccines that did not contain recombinant FHbp (control studies 1, 2, and 3, *P* = 0.17, 0.84, and 0.60, respectively). (C and D) Proportions of vaccinated subjects with any increase or decrease in anti-FH reactivity in paired sera. (C) Subjects given other vaccines in control studies 1, 2, and 3. Among 57 subjects, 51% had some increase in FH reactivity after vaccination (*P* > 0.99 compared to the expected value of 50%; see text). (D) Subjects given MenB-4C. Among 140 subjects with paired pre- and postimmunization sera, 67% showed increases in anti-FH reactivity after vaccination (*P* < 0.0001 compared to the expected percentage of 50% by chance alone; *P* = 0.036 compared to the percentage of control subjects represented in panel C).

Panel A of Fig. 2 shows the median change and 95% confidence interval in serum anti-FH reactivity after MenB-4C vaccination for each of the two vaccination schedules. We expect natural fluctuations and assay variability in serum OD_405_ values in the paired pre- and postimmunization sera. If these changes were truly random (i.e., not affected by vaccination), then the median difference between the respective pre- and postimmunization OD_405_ values would be 0. For both vaccination schedules, the observed median difference exceeded the hypothetical difference of 0 and the lower limits of the 95% confidence interval did not go below 0 (*P = *0.0001 and 0.028 compared to a theoretical value of 0 by one-sample *t* test, in the accelerated and standard vaccination schedules, respectively). Thus, the null hypothesis of no difference was rejected.

### Assays of stored sera from previous immunogenicity studies.

In the historical collections of stored paired pre- and postimmunization sera, there was a similarly small but statistically significant increase in serum anti-FH binding activity after MenB-4C vaccination but not after vaccination with other meningococcal vaccines that did not contain recombinant FHbp. For the MenB-4C historical sera, the geometric mean OD_405_ values were 0.47 preimmunization and 0.51 postimmunization (*P = *0.0024), and the lower limit of the 95% confidence interval of the median change in anti-FH reactivity did not go below 0 (*P = *0.007; Fig. 2B). For the MenC conjugate vaccine historical sera, the geometric mean preimmunization and postimmunization OD_405_ values were 0.59 and 0.56 (*P = *0.27). Thus, there was no trend for higher values in the postimmunization sera and the observed small decrease after vaccination was not statistically significant. For the two outer membrane vesicle (OMV) studies, the preimmunization geometric mean OD_405_ values were 0.49 and 0.52 and the respective postimmunization OD_405_ values were 0.53 and 0.53 (*P = *0.14 and 0.71, respectively). Further, the lower limits of the 95% confidence interval of the respective median changes in anti-FH reactivity in the paired pre- and postvaccination serum samples from the subjects immunized with the other meningococcal vaccines were below 0 (*P = *0.17 and 0.84 for the OMV vaccine studies and *P = *0.60 for the MenC conjugate vaccine study by Wilcoxon signed rank test comparing the median change to a hypothetical value of 0; Fig. 2B). Thus, we can reject the null hypothesis of no difference between pre- and postvaccination values for each of the two MenB-4C vaccination schedules used in the prospective study and for the historical MenB-4C study with paired serum samples available from each subject but not for the three historical serum collections from subjects with paired serum samples who were immunized with other meningococcal vaccines that did not contain recombinant FHbp.

We then aggregated the data for the two schedules used in the prospective MenB-4C study and historical MenB-4C study 1 and, separately, the data for the subjects given other vaccines that did not contain recombinant FHbp. If the changes in OD_405_ between the respective paired pre- and postimmunization sera were random, then the proportion of subjects with increases above 0 (or decreases below 0) by chance would be expected to be 50%. Among the 57 subjects given meningococcal vaccines that did not contain recombinant FHbp, 29 (51%) showed higher anti-FH values in postimmunization sera than in preimmunization sera, which could be explained by chance alone (*P > *0.99; Fig. 2C). In contrast, among the 140 subjects immunized with MenB-4C (prospective study and the historical MenB-4C study 1), 94 (67%) showed higher anti-FH reactivity in postimmunization then in prevaccination sera. This percentage is significantly higher than the 50% expected for chance alone (*P < *0.0001 compared with a theoretical proportion of 50% by Z test; Fig. 2D). The 67% proportion in the MenB-4C-vaccinated subjects also is significantly higher than the corresponding 51% proportion of the subjects given vaccines that did not contain recombinant FHbp (*P = *0.036, Fisher’s exact test; Fig. 2C and D). We interpret these results collectively as representing a weak immunogenic response to FH for the groups given MenB-4C but not for the groups given the other meningococcal vaccines.

**FIG 3:**
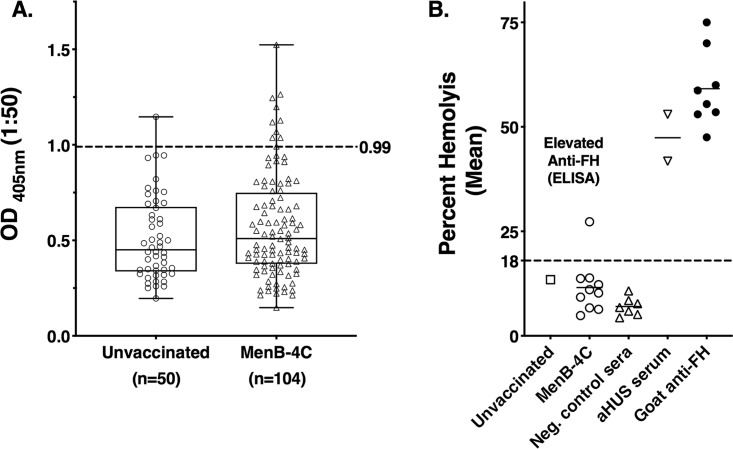
Serum anti-FH antibody reactivity of university students immunized with two doses of MenB-4C given in response to a meningococcal serogroup B campus outbreak. (A) Anti-FH ELISA. All sera were tested at a 1:50 dilution. The median serum anti-FH OD_405_ value determined for the unvaccinated students was 0.45 compared to 0.51 in sera obtained 1.5 to 2 months after immunization of students with two doses of MenB-4C (*P* = 0.23 by unpaired Mann-Whitney test). The boxes show the medians and quartiles, and the whiskers represent the ranges. The dashed line represents 2 SD above the mean (0.99). Elevated anti-FH reactivity was present in 1 of 50 unvaccinated students (2%) compared to 10 of 104 MenB-4C-vaccinated students (9.6%) (*P* = 0.105 by Fisher’s exact test). (B) Effect of serum FH on protection of sheep red blood cells from alternative-pathway-mediated hemolysis. Data are shown for the 11 sera from historical MenB-4C study 2 with elevated anti-FH reactivity by ELISA (1 unvaccinated and 10 vaccinated with MenB-4C; panel A). For the hemolytic assay, all sera were diluted 1:9, and the upper limit of normal lysis was 18% (see Materials and Methods). One of the 11 sera (from a MenB-4C-vaccinated subject) showed elevated hemolysis (27%), which was confirmed in an independent assay. Negative-control sera were from 7 unvaccinated adults with low anti-FH levels by ELISA. Control sera with abnormal FH function were from a patient with aHUS (patient C, Fig. 1) and a healthy human serum pool mixed (95:5) with a goat antiserum to FH. For the positive controls, each symbol represents a value in replicate assays.

Preimmunization serum samples were not available from the second historical MenB-4C study (Table 1), and we compared anti-FH in sera from vaccinated and unvaccinated students. For the 50 unvaccinated control students, the median serum anti-FH OD_405_ was 0.45, compared to 0.51 in sera obtained 1.5 to 2 months after immunization of 104 students given two doses of the MenB-4C vaccine (Fig. 3A; *P = *0.23 by unpaired Mann-Whitney test). High anti-FH reactivity (OD_405_ of >0.99, which was >2 SD above the mean for unvaccinated subjects) was present in 1 of 50 unvaccinated students (2%) compared to 10 of 104 MenB-4C-vaccinated students (9.6%, *P = *0.105 by Fisher’s exact test).

**TABLE 1:** Summary of prospective and historical immunogenicity studies providing sera for investigation of anti-FH reactivity^*a*^

Study	Location(s) of study	Study design	Demographics	Vaccine schedule	No. of subjects	Reference
MenB-4C prospective study	Quebec, Nova Scotia, and British Columbia, Canada	Paired pre- and 3 wks post-dose 2 immunization sera	High school and university students, 17–25 yrs of age	Schedule A, 2 doses at 0 and 3 wks; schedule B, 2 doses at 0 and 2 mos	58; 62	Langley (51)

Historical sera from previous immunogenicity studies						
MenB-4C						
Study 1	Northern California, USA, and Oxford, United Kingdom	Paired pre- and 4 wks postimmunization sera	Healthy adults 21–44 yrs of age	2 doses at 0 and 2 mos	20	Giuntini et al. (20)
Study 2	Northern California, USA	Sera from vaccinated and unvaccinated subjects (not paired)	College students	0 doses; 2 doses at 0 and 2 mos	50; 104	Lujan et al. (45)
OMV control						
Study 1	Oxford, United Kingdom	Paired pre- and 4 wks postimmunization	Healthy adults 18–50 yrs of age	3 doses at 0, 2, and 4 mos	20	Marsay et al. (30)
Study 2	Northern California, USA	Paired pre- and 3–6 wks postimmunization	Healthy adults 18–50 yrs of age	3 doses at 0, 1, and 2 mos	13	Plested et al. (29)
MenC conjugate control study 3	Northern California, USA	Paired pre- and 4 wks postimmunization	Healthy adults 18–50 yrs of age	1 dose	24	Vu and Granoff (28)

a For historical MenB-4C study 1 (20), 1 of the 20 subjects had predose and 1-month postdose 3 sera. Historical MenB-4C study 2 (45) did not have prevaccination sera. Control study 1 tested an investigational outer membrane vesicle (OMV) vaccine prepared from Neisseria meningitidis B strain H44/76 with constitutive expression of FetA (30). Ten of the 20 subjects received a dose of 25 μg, and 10 received a dose of 50 μg. Because the respective results from the two groups were similar, for the present study, the anti-FH reactivity data were combined. Control study 2 tested an investigational OMV vaccine from H44/76 using a dose of 50 μg (29). Control study 3 tested one dose of a serogroup C meningococcal conjugate vaccine prepared by Chiron Vaccines (28).

### Individual subjects with increases in serum anti-FH titers after vaccination.

**FIG 4:**
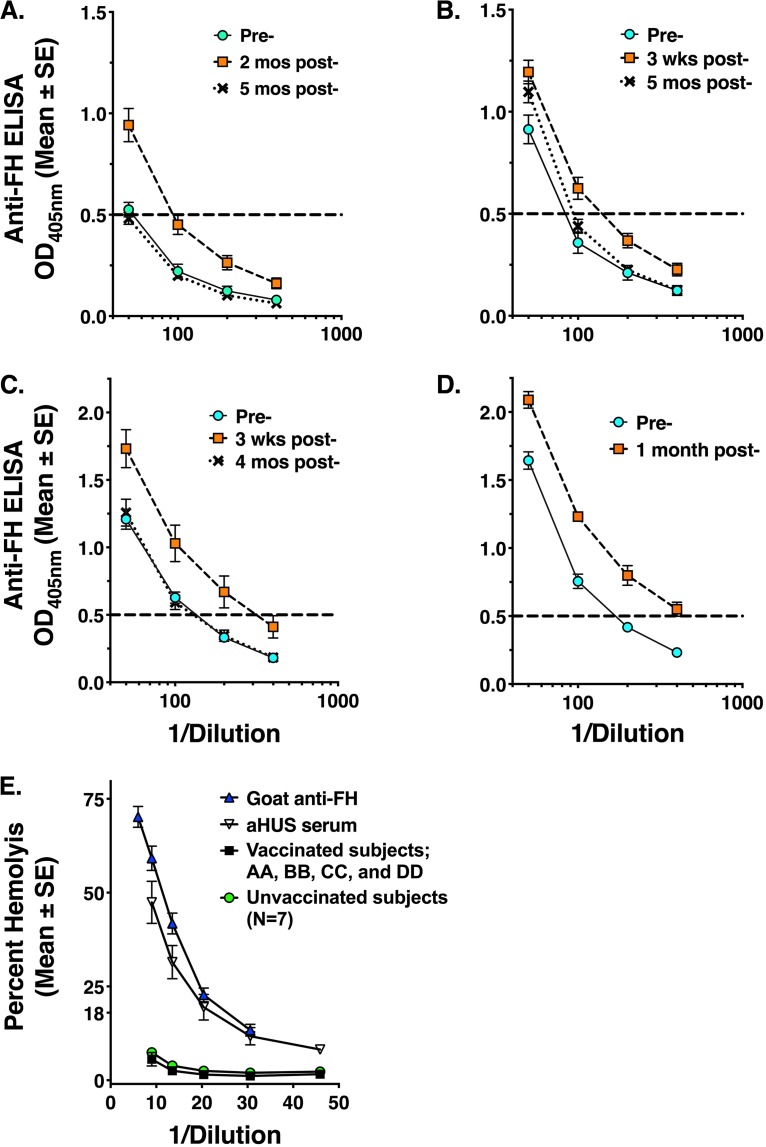
Individual subjects with increases in serum anti-FH reactivity after vaccination. (A to D) Four subjects had increases in serum anti-FH antibody titers 3 weeks to 2 months after vaccination (preimmune [Pre-]; light blue circles with solid lines), 3 weeks to 2 months postimmunization (orange squares with dashed lines), and 4 to 5 months postimmunization (black “X” symbols with dotted lines). Subjects AA, BB, and CC were immunized with MenB-4C (panels A, B, and C, respectively). Their titers returned to baseline (panel A and C) or to a level close to baseline (panel B) by 4 to 5 months. Subject DD (panel D) was immunized with an OMV vaccine without recombinant FHbp as part of historical control vaccine studies (Table 1). This subject did not have a subsequent postimmunization serum sample available to determine if the increased anti-FH antibody persisted. (E) Serum FH function as measured by alternative-pathway-mediated hemolysis. Data represent unvaccinated adult sera (*n* = 7), each with low anti-FH binding by ELISA (OD_405_ of <0.47 at a 1:50 dilution), and postvaccination sera (3 weeks to 2 months; subjects AA, BB, CC, and DD). Controls with abnormal FH function included a serum sample from a patient with aHUS and a human serum pool mixed with goat antiserum to human FH as described in the Fig. 3B legend. SE, standard error.

Among the 140 subjects vaccinated with MenB-4C with paired pre- and postimmunization serum samples (120 in the prospective study and 20 in the historical MenB-4C study 1), 3 subjects (2.1%) with low levels of anti-FH antibody in preimmune sera had elevated levels of postimmunization serum anti-FH antibody (>2 SD above the mean OD_405_ for unvaccinated subjects). The pre- and postimmunization serum anti-FH titers of these 3 subjects were tested using serial dilutions, and the anti-FH titers were calculated based on the serum dilution with an OD_405_ intercept of 0.5 in multiple replicate assays. For all three subjects (designated AA, BB, and CC in Fig. 4A, B, and C, respectively), the anti-FH titers were higher in the sera collected 3 weeks to 2 months postimmunization than in the respective preimmunization sera (*P < *0.01 for each subject by *t* test). By 4 to 5 months postimmunization (indicated with “X” symbols with dotted lines), serum anti-FH titers had returned to baseline in two subjects (AA and CC) and were close to baseline in the third subject (BB), and no serious adverse events were reported during this time period. One of the 57 subjects given other meningococcal vaccines (1.8%) had a 2.4-fold increase in anti-FH antibody titer at 1 month postimmunization (*P = *0.004, compared to preimmunization titer; Fig. 4D). This subject (designated DD) had been immunized with an OMV vaccine in a U.S. study and did not have a subsequent serum sample available to determine if the increased anti-FH antibody titer persisted beyond 1 month.

### Effect of anti-FH antibody on FH function.

Autoantibodies to FH can impair FH function, which is implicated in the pathogenesis of autoimmune atypical hemolytic uremic syndrome (16–18). We therefore tested sera from the four subjects with elevated anti-FH antibody titers at 3 weeks to 2 months postimmunization (subjects AA, BB, CC, and DD; Fig. 4) for the ability of their serum FH to protect sheep red blood cells from hemolysis mediated by the alternative pathway (AP) (19). Normal serum complement does not lyse sheep red blood cells efficiently under the conditions used because serum FH controls the activation of the AP and blocks the formation of the membrane attack complex. If the FH in the test serum were less functional because of a genetic polymorphism or autoantibody to FH, there would be more lysis of the sheep cells than in serum with normal FH function. Thus, a low percentage of hemolysis implies the presence of fully functional FH whereas excessive lysis indicates less inhibition and dysfunctional FH (or low levels of FH). Panel E of Fig. 4 shows mean hemolytic data for 7 negative-control sera from unvaccinated adults with low levels of serum anti-FH antibody binding by ELISA (OD_405_ of <0.45 at a 1:50 serum dilution) and the corresponding data from testing postimmunization sera from subjects AA, BB, CC, and DD with elevated anti-FH titers. All 11 sera had normal FH function. In contrast, a serum sample from a patient with aHUS (patient C; Fig. 1) had impaired FH functional activity as evidenced by excess hemolysis, as did normal human serum mixed with a goat antiserum to FH (see Materials and Methods). There were insufficient quantities of sera from the two patients with autoimmune aHUS (patients A and B) for testing in the hemolytic assay.

We also tested FH function for the 11 stored sera with elevated anti-FH reactivity from MenB-4C historical study 2 (1 unvaccinated subject and 10 vaccinated subjects). One sample from a vaccinated subject showed impairment of the ability of serum FH to protect sheep red blood cells from AP-mediated hemolysis (27% hemolysis; Fig. 3B). This result was confirmed in a second independent assay.

## DISCUSSION

Both of the licensed MenB vaccines contain recombinant FHbp, which in previous studies in human FH transgenic mice and infant macaques elicited transient cross-reactive antibodies to human FH (9, 11, 52). However, it is not known if autoantibodies to FH are elicited in humans immunized with MenB vaccines. To address this issue, we conducted a prospective multicenter immunogenicity study of the MenB-4C vaccine and found small but statistically significant increases in serum antibody reactivity to FH at 3 weeks after vaccination using either of the vaccination schedules tested. The findings were confirmed in analysis of the anti-FH reactivity of stored paired pre- and postimmunization sera from a previous MenB-4C immunogenicity study in adults (MenB-4C study 1) (20) but not in analysis of the anti-FH reactivity of stored sera from three groups of adults given other meningococcal vaccines that did not contain recombinant FHbp. Conceivably, binding of host FH to the recombinant FHbp vaccine antigen in MenB-4C leads to conformational changes in FH, which are recognized as “foreign” by the immune system. The immunogenicity of FH in the complex also might be enhanced by adjuvant activity from the OMV component of the MenB-4C vaccine and/or the aluminum hydroxide adjuvant.

While statistically significant, the average increases in serum anti-FH OD_405_ following administration of the MenB-4C vaccine containing recombinant FHbp were much lower than in serum from patients with autoimmune aHUS, and the increased postimmunization values are within the range measured in unvaccinated sera. Overall, these small average increases in anti-FH after vaccination are therefore unlikely to pose an increased risk of anti-FH autoimmune diseases; however, they suggest that MenB-4C has the potential to perturb the ability of the immune system to recognize host FH.

Serum autoantibodies to FH have been found naturally in a small proportion of healthy persons. In one study, 1% of blood donors with a mean age of 43 years (range, 18 to 68) and 8% of older subjects with a mean age of 78 years (range, 48 to 92) had serum anti-FH antibody titers that were >2 SD above the mean (21). In most cases, the antibodies did not appear to have deleterious effects (22). However, anti-FH autoantibodies are implicated in the pathogenesis of certain human diseases involving complement dysregulation such as autoimmune aHUS (17, 18, 23–25) and C3G (17, 26).

FH consists of 20 domains, referred to as short consensus repeats (SCR). In autoimmune aHUS, the anti-FH autoantibodies are reported to be primarily directed at SCR 19 and 20 in the C-terminal region of the molecule (16, 27), which bind self surfaces and the C3d portion of C3b. Antibodies directed at this region can decrease FH function as measured in hemolytic assays (16–18). In autoimmune aHUS, the anti-FH antibodies have been reported to decrease FH function (18). With impaired ability of FH to downregulate complement activation (17, 18), unchecked complement activation can lead to onset of renal disease and hemolytic anemia characteristic of aHUS. While C3G also is associated with serum autoantibodies to FH (17), the antibodies are reported to be of lower avidity for FH than in patients with autoimmune aHUS (23), to be directed at different FH epitope specificities located in the N-terminal region (23), and typically not to affect FH functional activity as measured in the hemolytic assay (17). Nevertheless, the anti-FH autoantibodies appear to have some functional consequences with respect to progression of renal disease in patients with C3G, perhaps in concert with other antibodies such as C3 nephritic factor (17).

In our prospective study, three MenB-4C-vaccinated subjects showed increases in serum anti-FH binding after vaccination that approached the anti-FH titers present in sera from two patients with autoimmune aHUS. However, the increased titers after vaccination were transient and returned to baseline or near baseline by 4 to 5 months. Further, none of the sera with elevated titers had impaired FH function measured in the hemolytic assay. In historical MenB-4C study 2, 10 MenB-vaccinated college students with stored postimmunization sera (9.6%) had elevated anti-FH antibody at 6 weeks to 2 months postimmunization versus 1 (2%) unvaccinated student. Important limitations of this study were a lack of preimmunization sera to determine whether the anti-FH antibodies were present before vaccination and a lack of additional follow-up sera to determine antibody persistence. However, only 1 of the 11 serum samples with elevated anti-FH antibody activity from vaccinated students had low FH function in the hemolytic assay, which suggested that most of the subjects with elevated serum anti-FH antibody reactivity had minimal risk of developing autoimmune aHUS. However, the risk of C3G in this population is not known since serum anti-FH antibodies in patients with C3G are reported not to affect the level of FH function measured by the hemolytic assay (17).

As noted above, increased serum anti-FH reactivity was not observed in stored paired pre- and postimmunization sera from three other historical studies of other meningococcal vaccines that did not contain significant levels of recombinant FHbp (28–30). Two of these studies investigated OMV vaccines prepared from group B strain H44/76, which naturally expresses FHbp. Interestingly, one individual given an OMV vaccine exhibited a significant increase in serum reactivity to FH (Fig. 4D). OMVs are treated with detergents to decrease endotoxin content. The treatment also removes other detergent-soluble molecules, including FHbp, which is a lipoprotein, and FHbp is reported to be a very minor protein in the detergent-extracted outer membrane vesicle (dOMV) prepared from strain NZ98/254 (<0.045 μg per 25-μg dose [31]) or from strain H44/76 (0.01 to 0.025 μg per 25-μg dose [32, 33]). Whether this small amount (less than 0.05 μg per 25-μg human dose) is sufficient to recruit FH and elicit anti-FH antibody response is not known. Our data indicated that the subjects given OMV vaccines did not as a group show increases in anti-FH OD_405_ after vaccination (*P = *0.14 and 0.71). Thus, there was no statistical evidence that vaccination evoked antibody to FH, which is consistent with our hypothesis that the potential for eliciting anti-FH antibodies is highest when FHbp is present in high amounts (i.e., 50 μg of the recombinant fusion protein in MenB-4C). While it is not possible to draw conclusions from a single case of elevated anti-FH titers in an OMV-vaccinated subject, there is a possibility that host FH complexed with residual FHbp in the OMV might have been responsible.

As of February 2018, an estimated 20 million doses of MenB-4C had been distributed worldwide (https://www.gsk.com/en-gb/media/press-releases/gsk-s-meningitis-b-vaccine-bexsero-receives-breakthrough-therapy-designation-from-us-fda-for-prevention-of-invasive-meningococcal-disease-in-children-2-10-years-of-age/; accessed 20 March 2019). To date, there have been no published reports suggesting elevated incidences of autoimmune aHUS or C3G after MenB-4C vaccination. However, four cases of nephrotic syndrome were identified recently in children ages 2 to 5 years who had been vaccinated with MenB-4C during a mass immunization campaign in Quebec, Canada (34). The nephrotic syndrome was considered to be idiopathic based on therapeutic responses to steroid therapy. However, none of the cases had a biopsy performed or serum anti-FH antibody level determined, and one of the cases relapsed and required long-term immunosuppression therapy. Given the relative rarity of nephrotic syndrome in the population, with an estimated annual incidence of 17.7 per 100,000, the four cases were considered by the authors to represent “a potential vaccine safety signal.”

In summary, the lack of reports of autoimmune aHUS or C3G is reassuring, but these diseases are rare in the population and onset is likely to be delayed for months or years beyond vaccination and may require a secondary trigger (35). Therefore, an association with vaccination will be difficult to ascertain (36). Also, it is possible that only certain subgroups of the population are at increased risk of developing anti-FH related autoimmune diseases, such as persons with preexisting autoimmune diseases or healthy persons with a deficiency of complement factor H (CFH)-related proteins such as occurs in ∼ 6% of the population (37), and are associated with autoantibodies to FH and aHUS (24, 26, 38, 39). The risk of these diseases also may be heightened in the presence of rare variants in the FH gene at functional sites or in the context of naturally low serum FH levels (40–42) if these persons should also acquire anti-FH autoantibodies. Thus, there remains a need for continued surveillance for diseases associated with anti-FH autoantibodies in larger populations and possibly for performing case-control studies in immunized and unimmunized persons to determine relative risk.

Finally, our studies of serum anti-FH reactivity were limited to adults immunized with the recommended two doses of MenB-4C. Similar studies are needed in infants and children given the recommended two-dose or three-dose schedule used in Europe and in adults immunized with the MenB-FHbp vaccine (Trumenba), which contains two lipidated FHbp antigens (43, 44). Studies also are needed on serum anti-FH antibody in adults given booster doses of MenB-4C or MenB-FHbp, since serum bactericidal titers after the recommended 2-dose or 3-dose schedules can decline within a year to levels below those considered protective (43, 45, 46), and booster doses may be needed, which may also boost anti-FH reactivity. This is particularly true for adults at increased risk of developing meningococcal disease such as laboratory workers with occupational exposure to meningococci (47) and patients with aHUS or paroxysmal nocturnal hemoglobinuria who are undergoing complement inhibition therapy (48–50).

## MATERIALS AND METHODS

### Human serum samples.

Serum samples were obtained from a prospective, randomized, observer-blinded Canadian Immunization Research Network (CIRN) clinical trial with the primary aim to compare serum bactericidal antibody responses to two different MenB-4C vaccination schedules in adolescents and young adults aged 17 to 25 years. An additional prespecified aim was to measure serum anti-FH antibodies. In brief, subjects were randomly assigned either to the standard two-dose MenB-4C schedule given intramuscularly (i.m.) at 0 and 8 weeks or to an accelerated two-dose schedule at 0 and 3 weeks. Hepatitis A vaccine was given i.m. at 3 or 8 weeks, respectively, to maintain blinding of the investigators to the MenB-4C vaccination schedule. A total of 120 subjects completed the vaccination schedules and provided the requisite serum samples, including 62 with the standard schedule and 58 with the accelerated schedule. The primary analysis of anti-FH reactivity was performed on sera obtained immediately before dose 1 (preimmunization) and 3 weeks post-MenB-4C dose 2. In subjects with elevated serum anti-FH reactivity (>2 SD above the mean anti-FH antibody level in unvaccinated sera) at 3 weeks to 2 months postvaccination, we also measured anti-FH titers in follow-up sera obtained at 4 to 5 months after dose 2.

To supplement the data from the prospective Canadian study, we also measured anti-FH reactivity in stored serum samples from five previously described immunogenicity studies (Table 1). These included two studies performed in college students or hospital personnel immunized with MenB-4C (20, 45) and three studies in young adults given other meningococcal vaccines, which, unlike MenB-4C, do not contain recombinant FHbp. The other meningococcal vaccines consisted of outer membrane vesicle (OMV) vaccines (29, 30) or a serogroup C conjugate vaccine (28) (Table 1). For the studies of the historical stored sera (20, 28–30), we assayed paired preimmunization and 3-week to 1-month postimmunization serum samples that had been deidentified, and the results were not linked to the respective identities of the individuals.

### Serum anti-FH antibody.

Total (IgG, IgM, and IgA) levels of serum anti-FH reactivity were measured by ELISA in sera diluted 1:50 using a published protocol (15) with minor modifications. In brief, we used U-bottom ELISA plates (Immulon 2HB; Thermo Scientific) in which a mixture of 2 μg/ml of purified human complement factor H (Complement Technologies) and phosphate-buffered saline (PBS) was placed and incubated the plates overnight at 4°C. After washes were performed, 5% milk–PBS was added to each well (blocking step) and incubated for 2 h at room temperature. Except where noted, all serum samples were assayed at a 1:50 dilution (dilution buffer; PBS, 1% bovine serum albumin [BSA], 0.1% Tween 20, 0.01% NaN_3_). Replicate 100-μl samples were added to wells in separate plates and incubated for 3 h at room temperature. Bound antibody was detected by the use of goat anti-human IgG, IgM, and IgA (H+L) secondary antibody conjugated with alkaline phosphatase (Invitrogen) (1:10,000). Paired pre- and postimmunization sera were assayed for anti-FH binding in parallel on the same plates. The anti-FH reactivity of each serum sample was expressed as the mean of the OD_405_ values of the replicate determinations. Selected sera with elevated anti-FH levels at a 1:50 dilution were reassayed at a series of dilutions, and anti-FH titers were assigned based on the serum dilution with an OD_405_ intercept value of 0.5 (see, for example, Fig. 1B).

### Anti-FH functional activity.

To determine the effect of anti-FH antibody on FH function, we used a hemolytic assay that measures the ability of serum FH to protect sheep red blood cells from alternative-pathway-mediated hemolysis. In the presence of low functional FH activity, the sheep erythrocytes undergo complement-mediated lysis (measured at OD_415_ by release of hemoglobin using a spectrophotometer). In brief, dilutions were performed with gelatin veronal buffer (GVB^−/−^) (also referred to as GVB^o^ without Ca^++^ or Mg^++^; Complement Technologies), which was supplemented with final concentrations of 0.01 M EGTA and 0.007 M MgCl_2_. Serial dilutions of the test serum (50 μl) were mixed with equal volumes (50 μl) of sheep erythrocytes (Colorado Serum Company) and a 1:10 dilution of normal serum that had been depleted of FH and factor D (Complement Technologies). The reaction mixture was incubated at 37°C for 60 min, the reaction was stopped by the addition of 2 ml of cold saline solution, and hemolysis was calculated by measuring hemoglobin released into the supernatant based on OD_415_. Control reactions included buffer only (0% lysis) or water only (100% lysis). Percent hemolysis was calculated as follows: percent hemolysis = 100 * {[test serum OD − buffer OD (0% lysis)]/[water OD (100% lysis) − buffer OD (0% lysis)]}. As a positive control, a normal human serum pool was tested that had been mixed with a goat antiserum to human factor H that blocks FH function (Quidel; A312) [5% (vol/vol)]. Aliquots of the pooled normal serum and goat anti-factor H antibody mixture were stored frozen at −70°C and were thawed only once. In assays of sera from 88 healthy adults, the upper limit of hemolysis using a 1:9 dilution of the serum was 18% (2 SD above the mean percent hemolysis).

### Statistical analyses.

Our null hypothesis was that, compared to preimmunization values, the average change in OD_405_ after immunization is expected to be 0 since in the absence of a vaccine effect, the proportion of subjects with increases above 0 (or decreases below 0) by chance is 50% (these can be explained by minor variability in the assay or fluctuations in natural levels of anti-FH reactivity). For the paired pre- and postimmunization sera, we compared the respective means of the log_10_-transformed OD_405_ values by the use of paired *t* tests. Differences in OD_405_ values (postimmunization – preimmunization) were evaluated using a Wilcoxon signed-rank test comparing column medians to a hypothetical value of 0. The proportions of two groups were compared using a Fisher’s exact test or a Z test as specified in the text. All *P* values were calculated based on two-tailed hypotheses.

### Ethical approvals.

For the prospective MenB-4C CIRN study in Canada, all subjects provided signed written informed consent after the nature and possible consequences of the study had been fully explained. The protocol and studies were approved by the participating site Research Ethics Boards. Further details of the study, which was conducted according to International Conference on Harmonisation-Good Clinical Practice (ICH-GCP) guidelines, are available at ClinicalTrials.gov (registration no. NCT02583412). Assay of the serum samples for anti-FH also was approved by the UCSF Benioff Children’s Hospital Oakland Institutional Review Board (IRB) of the University of California, San Francisco (UCSF). The historical control sera were from five previously published studies (Table 1). Anti-FH reactivity was measured on coded sera, which were deidentified. MenB-4C study 1 (20) was approved by the Institutional Review Board of UCSF Benioff Children’s Hospital Oakland, Oakland, CA (IRB no. 2015-055 and 2016-24), and by the National Research Authority Ethical Committee, Bristol, United Kingdom (15/SC/0172, protocol 2014-12). MenB-4C study 2 (45) was approved by the Institutional Review Board of UCSF Benioff Children’s Hospital Oakland (IRB no. 2016-044). Approval of control study 1 (OMV vaccine [30]) was granted by National Research Ethics Service (NRES) Oxford A (12/SC/0023), Oxford, United Kingdom. Approval of control study 2 (OMV vaccine [29]) was granted by the Institutional Review Board of UCSF Benioff Children’s Hospital Oakland (IRB no. 2004-018). Control study 3 (meningococcal C conjugate vaccine [28]) was approved by the Institutional Review Board of UCSF Benioff Children’s Hospital Oakland (IRB no. 2003-16).
